# A quality improvement initiative for patients with chronic kidney disease to promote their smoking cessation

**DOI:** 10.18332/tid/170626

**Published:** 2023-10-09

**Authors:** Cheng-Hsu Chen, Tzu-Mei Lin, Su-Chi Hung, Ming-Ju Wu, Shang-Feng Tsai

**Affiliations:** 1 Division of Nephrology, Department of Internal Medicine, Taichung Veterans General Hospital, Taichung City, Taiwan; 2 Department of Life Science, Tunghai University, Taichung City, Taiwan; 3 Department of Post-Baccalaureate Medicine, College of Medicine, National Chung Hsing University, Taichung City, Taiwan; 4 Tissue Engineering and Regenerative Medicine, College of Medicine, National Chung-Hsing University, Taichung, Taiwan

**Keywords:** quality improvement (QI), smoking cessation, Ottawa model for smoking cessation (OMSC), chronic kidney disease, payment-for-performance (P4P)

## Abstract

Cigarette smoking is a critical issue in caring for patients of chronic kidney disease (CKD). However, there is no routine care program designed for combining both smoking cessation and CKD care.

The process of our quality improvement (QI) collaboration used data under our routine payment-for-performance for pre-end-stage renal disease (P4P Pre-ESRD) in Taichung Veterans General hospital from 2020 to 2022. We share our experience with a QI project that integrates the Ottawa model for smoking cessation (OMSC) with the Pre-ESRD care program as part of routine CKD care. The electronic health information systems were improved to reduce workload of medical staff. The number was more for both qualified CKD educators and nephrologists for smoking cessation.

The access and availability for smoking cessation were immediate and convenient for patients. Specifically, all the actions were performed by CKD educators, with nephrologists overseeing the process in routine care. By combining OMSC with the Pre-ESRD program, we were able to provide smokers with satisfactory access and availability to smoking cessation services within our healthcare facility. The smoker cases found were more in number (206 in 2020, 344 in 2021, and 421 in 2022). Before the integrated OSTC-Pre-ESRD program (in 2020), the proportion of smokers was 3.88%. After implementing the integrated program, smokers increased significantly on a yearly basis (9.69% in 2021 and 9.82% in 2022). Finally, case numbers of on-site smoking cessations increased significantly after implementing the integrated system (0 in 2020, 17 in 2021, and 20 in 2022). All CKD patients for smoking cessation were also more (8 in 2020, 46 in 2021, and 38 in 2022).

After implementing the QI program, focusing on the integrated OMSC-Pre-ESRD program, we found more smokers undergoing smoking cessation. This QI program highlighted the importance of better access and availability for smoking cessation.

## INTRODUCTION

According to the World Health Organization, the tobacco epidemic is one of the biggest public health threats worldwide. It causes the death of more than 8 million people every year. In 2020, over 20% of the global population used tobacco. Smoking is widely recognized as being associated with cardiovascular diseases, cancer, and chronic kidney disease (CKD)^1^. Smoking even a small number of cigarettes per day, up to 10 over a lifetime, increases the risk of death compared to never smokers^2^. However, quitting smoking can provide substantial benefits for individuals who smoke.

Smoking is believed to be associated with the development of CKD^3,4^. In a recent meta-analysis comprising 15 prospective cohort studies and including 65064 incident CKD cases, there is substantial evidence supporting cigarette smoking as an independent risk factor for incident CKD^5^. This evidence suggests a causal effect^6,7^. Therefore, smoking cessation can reduce proteinuria and the incidence of end-stage renal disease (ESRD)^8-10^. Despite the existing evidence, there is currently no specific guideline available for smoking cessation in CKD patients^11^. The methods for quitting smoking during standard CKD care are poorly studied, and most protocols are based on extrapolated evidence from the general population.

The Ottawa Model for Smoking Cessation (OMSC) is a well-known approach to quitting smoking^12^. It is a quality improvement program for smoking cessation based in Ontario. The OMSC utilizes an evidence-based approach that can be implemented in busy primary care practices, thereby improving the chances of successfully quitting smoking. Notable features of this program include a partnership with a smoker’s helpline, support from electronic medical records, motivation strategies for patients who are not yet ready to quit, and the OMSC Primary Care Quality Improvement Plan Toolkit. The 3As model (ask, advise, and act) used in this program represents a practice change process and an evidence-based protocol for smoking cessation. The OMSC has been widely implemented for both inpatients^13^ and outpatients^14^, showing good long-term abstinence rates. However, it has not been specifically implemented for patients with CKD. In this study, we share our experience with a quality improvement (QI) project that integrates the OMSC with the Pre-ESRD care program as part of routine CKD care.

## METHODS

### Nationwide pay-for-performance (P4P) program is available for Pre-ESRD care

The prevalence of CKD exceeds 10%, representing a significant global health burden^15^. In Taiwan, the national prevalence of CKD is as high as 11.93%^16^ with the incidence of ESRD ranking among the top three worldwide, and its prevalence being the highest in a study conducted a decade ago^17^. According to a recent study in 2021, Taiwan has the highest incidence of ESRD globally^18^. The cost of dialysis accounts for up to 8% of the national medical expenditure^19^. In Taiwan, a nationwide pay-for-performance (P4P) program is available for Pre-ESRD care education^20^. Since its inception in 2006, this program has provided a promising approach through value-based purchasing, incentivizing renal indicators to improve healthcare quality and disease prognosis for patients in CKD stages 3b to 5^19,21^. This multidisciplinary care program covers over 100000 patients with late-stage CKD, and the enrollment rate was more by almost 100% from 2010 to 2018^22^. Within the Pre-ESRD P4P care program, nephrologists and CKD educators are required to collect data on smoking status. The P4P Pre-ESRD care program has been shown to lower the risks of both dialysis initiation and death^19,21^ while improving patient outcomes, such as lower hemodialysis rates, fewer hospitalization events, and better overall survival^23^. Therefore, we conducted this study to examine the effect of smoking cessation within the Pre-ESRD P4P care program.

### Features of Pre-ESRD P4P care program in Taichung Veterans General Hospital (TCVGH)

Our study was conducted at Taichung Veterans General Hospital (TCVGH), a medical center with 1500 beds and approximately 5500 employees. TCVGH is renowned for providing safe and high-quality medical services, equipped with advanced facilities and comprehensive training programs. It serves as a referral hospital in Taiwan, with the highest case-mix index reflecting the complexity and risk of diseases and the difficulty in treatment. Additionally, TCVGH has the largest number of CKD cases enrolled in care programs across the nation, including both early CKD and Pre-ESRD cases. For instance, in 2018 alone, more than 10000 patients were enrolled in our care system. Cumulatively, over 13000 patients with CKD have been enrolled in our care system to date. In this study, our focus was on smoking cessation within the Pre-ESRD P4P care program specifically targeting patients with estimated glomerular filtration rate (eGFR) below 45 mL/min/1.732 m^2^.

### Quality improvement (QI) study for smoking cessation in Pre-ESRD patients in TCVGH from 2020 to 2022

We present the comprehensive process of our quality improvement (QI) collaborative efforts, specifically focusing on smoking cessation data collected from CKD patients from 2020 to 2022. We describe the steps involved in implementing and assessing the outcomes of our QI initiative, including the number of smoking cessation cases and the involvement of qualified educators and nephrologists. Our efforts in smoking cessation reform began within our institute in 2020 as part of a broader QI initiative targeting patients with various chronic diseases such as chronic obstructive pulmonary disease, diabetes mellitus, congestive heart failure, coronary artery disease, and CKD. Data collection took place over a period of three consecutive years, from 2020 to 2022, with the QI projection specifically centered around CKD patients starting in 2021. To enhance our smoking cessation efforts, we integrated the Ottawa model for smoking cessation (OMSC) into our existing Pre-ESRD P4P care program and evaluated the outcomes, including the number of smoking cessation cases and the involvement of qualified educators and nephrologists.

### Enlisting a core change team

The core change team for this QI project was established in 2020, led by the hospital superintendent and the executive secretary, who is a physician. The core team for smoking cessation within our institute included representatives from various departments, including internal medicine, family medicine, surgery, pharmacology, nursing, medical affairs and planning, and laboratory medicine. The primary objective of this core team was to actively promote and facilitate smoking cessation among patients within our institute.

### Stakeholder mapping

**Figure 1:**
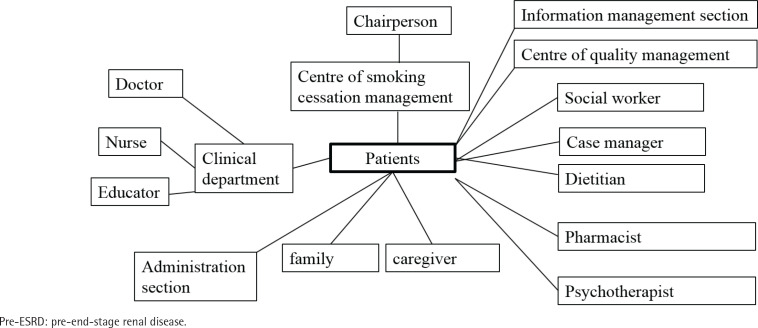
Stakeholder map for smoking cessation in pre-ESRD group in 2020. The quality of smoking cessation is shown at the center of the map, surrounded by various stakeholder groups

We created a stakeholder map (Figure 1) to identify key participants in our QI project. The QI project in this study shares similarities with our previously published study on patient support groups^24^. The target population consisted of patients already enrolled in our Pre-ESRD P4P care program at the outpatient department of nephrology. Stakeholders were categorized into different groups, including doctors, patients, caregivers, and hospital leaders, and their inter-group relationships were outlined. The central focus of this stakeholder map was the quality element of the project. The chairperson of the team was the hospital superintendent, and the leader was the chief of nephrology. Specialists from the Quality Management department and other secondary executives served as technical experts who were well-versed in the smoking cessation process. Team members were selected from various areas of the healthcare system, with doctors, educators, and patients/caregivers playing pivotal roles in the project.

### Action plans of QI for smoking cessation in patients with Pre-ESRD

The action plan following the PDSA (Plan, Do, Study, Act) cycle is summarized in Table 1. In 2021, our initial step involved screening the number of smoker patients currently enrolled in our Pre-ESRD P4P care program. The inclusion criteria for the Pre-ESRD P4P care program were patients in CKD stages 3b to 5. As part of the multidisciplinary care, a smoking status checkup was conducted. However, smoking cessation was not a mandatory requirement for participants in this care program. After implementing the smoking cessation quality improvement QI intervention, we began surveying the motivation for smoking cessation and identifying the major obstacles faced by patients. Based on feedback from current smokers in the Pre-ESRD stage, patients expressed a strong desire to quit smoking. However, they did not feel the need to wait for education or a prescription before initiating their smoking cessation efforts. This finding highlighted the importance of promoting accessibility and availability of smoking cessation resources for these patients.

**Table 1:** The action plan of PDSA (plan, do, study and action) in 2021 for current smoker patients in our Pre-ESRD P4P care program

**Plan**	Smoking cessation is vital for caring CKD patients. However, the case numbers of smoking cessation in our CKD care are always very limited. We initiated this qualify improvement program to increase case numbers of smoking cessation in patients with renal dysfunction. The identification of smoking patients is already done our regular Pre-ESRD P4P care program. However, patients cannot wait too long for smoking cessation even with motivation.
**Do**	We started this QI since 2021. We plan to integrate to OTSC into our regular Pre-ESRD care program. We encouraged our CKD care team members (nephrologists and educators) to be qualified for smoking cessation. We also created a dashboard to monitor the status. Smart and electronic medical record systems for prescription and follow-up were implemented for our nephrologists and educators.
**Study**	In our regular Pre-ESRD care system, the case numbers of Pre-ESRD were more. More educators and nephrologists were qualified for smoking cessation. As for smoking cessation, the case numbers of identified smoking also increased. More patients joined the OMSC-pre-ESRD care program.
**Action**	After this integrated OMSC-Pre-ESRD P4P care program, there is no need for referral in any process. This indeed improve the accessibility for smoking cessation in patients with renal dysfunction.

Pre-ESRD P4P: pay-for-performance (P4P) program for Pre-end-stage renal disease care education. CKD: chronic kidney disease. QI: quality improvement. OMSC: Ottawa model for smoking cessation.

The QI program was subsequently initiated with the objective of enhancing access and availability of smoking cessation services. To achieve this, we encouraged our CKD educators to undergo training and certification in smoking cessation techniques. Our nephrologists also acquired the necessary skills and certification to provide smoking cessation support. Additionally, the concept of OTSC (Onsite Education and Prescription) was integrated into our regular daily Pre-ESRD care program. This approach eliminated the need for smokers with Pre-ESRD status to be referred to external educators or physicians located in separate buildings. Following the planning phase, we implemented the action plan from 2021 to 2022 to assess the impact of our QI initiative on smoking cessation among these patients.

### Improved access and availability for smoking cessation for patients with Pre-ESRD

**Figure 2:**
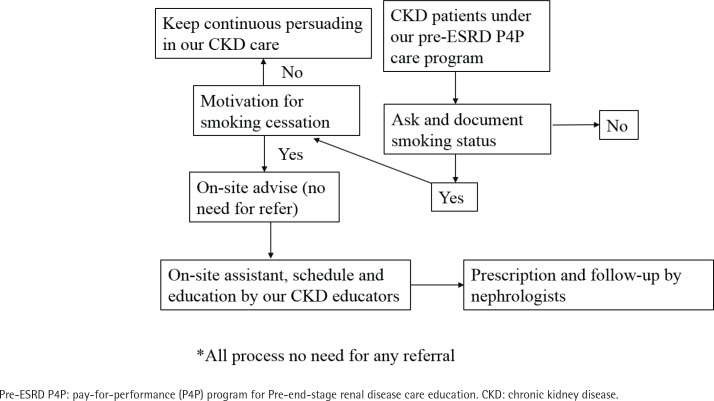
Integrated smoking cessation model in our regular pre-ESRD care program from 2020 to 2022

The action plan was designed to enhance access and availability of smoking cessation services for patients with Pre-ESRD. To achieve this, we integrated the OTSC approach with the Pre-ESRD P4P care program, as illustrated in Figure 2. Within our regular Pre-ESRD care program, we included the assessment of patients’ smoking status as a standard procedure during their visits. This integration did not impose an additional burden on CKD education. In the nephrology outpatient clinic, nephrologists and CKD educators inquired about patients’ smoking habits and their motivations to quit smoking. For those lacking motivation, we continued to follow up and employed strategies to increase their motivation. If patients expressed a desire to quit smoking, our CKD educators provided on-site advice, assistance, and scheduled support. Nephrologists were also able to prescribe medication for smoking cessation during the outpatient visit. Importantly, this entire process took place within the same healthcare facility, eliminating the need for patients to seek assistance elsewhere. As a result, access and availability for smoking cessation services were immediate and convenient for these patients.

### Integrated Ottawa model for smoking cessation for patients of Pre-ESRD

We successfully integrated the OMSC into our standard Pre-ESRD P4P care program, as outlined in Table 2. The original OMSC framework involved the following components: ask, document, advise, refer, and act. In our implementation, these procedures were executed by various healthcare professionals, including triage nurses, exam room escorts, medical doctors, pharmacologists, and nurses. However, we streamlined and integrated all these actions into our regular Pre-ESRD care program. Our CKD educators and nephrologists took responsibility for conducting all the actions within the integrated OMSC-Pre-ESRD program. Specifically, all the actions were performed by our CKD educators, with nephrologists overseeing the process in our outpatient clinic. By combining the OMSC with the Pre-ESRD program, we were able to provide smokers with satisfactory access and availability to smoking cessation services within our healthcare facility.

**Table 2:** The 3As model (ask, advise, and act) for implementing the Ottawa model for smoking cessation (OMSC) in patients with ESRD (integrated OSMC into our regular Pre-ESRD P4P care program)

*OTTAWA model*	*Associated medical staff in OTTAWA models*	*Implementation in our Pre-ESRD care*	*Associated medical staff in our models*
Ask and document smoking status	Triage nurse/exam room escort	Integrated questionnaire and asking in our Pre-ESRD care program	Educator
Advise and refer	Medical doctor and nurse practitioner	Both nephrologist and educators can initiate this program for smoking cessation	Nephrologists and educators
Act (quick plan visits and follow-up)	Nurse practitioner, pharmacologist, and registered nurse	Assistant and arrangement by our educators, prescription and follow-up from nephrologist. All integrated in our regular pre-ESRD care	Nephrologist and educators

Pre-ESRD P4P: pay-for-performance (P4P) program for Pre-end-stage renal disease care education.

### Increasing access and availability to smokers by medical staff

In addition to enhancing access and availability of smoking cessation services for patients, we implemented improvements to our electronic health information system to support the integrated OTSC-Pre-ESRD program. These enhancements proved beneficial, as CKD educators and nephrologists readily engaged in smoking cessation activities without experiencing an additional workload on top of their regular Pre-ESRD care duties. Supplementary file Figure S1 displays the smoking cessation dashboard, which allows us to screen and filter patients based on dates or outpatient clinics. This feature enables us to identify the smoking status of patients during their clinic visits and track their progress in smoking cessation. Moreover, Supplementary file Figure S2 demonstrates how CKD educators can evaluate the smoking status of their patients within the CKD education system. These improvements in the electronic health information system played a vital role in bolstering the motivation of CKD educators and nephrologists to prioritize smoking cessation. With efficient access to patient information, all medical staff could provide effective care for smokers without incurring additional burdens.

## DISCUSSION

### Improving smoking cessation in patients with Pre-ESRD

The number of pre-ESRD cases showed an increase from 2020 to 2022 (3079 in 2020, 3550 in 2021 and 4285 in 2022), both in terms of the cumulative count and the number of patients under active follow-up, who had not yet reached mortality or dialysis (Table 3). Following the implementation of the integrated system, the number of qualified CKD educators were more (2 vs 0). In 2020, there were no qualified educators, but after 2021, there were two. Similarly, the number of qualified nephrologists for smoking cessation were also more (4 vs 1). In 2020, there was only one qualified nephrologist, but after 2021, there were four.

**Table 3:** Case numbers of our Pre-ESRD P4P program, patients with smoking, and qualified educator and nephrologist for smoking cessation in 2020, 2021 and 2022

*Year*	*Case numbers of Pre-ESRD P4P program n*	*Case numbers of smoking n (%)*	*All CKD patients for smoking cessation n (%)*	*Patients join OMSC-Pre-ESRD program (on-site) n (%)*	*Qualified educators for smoking cessation (N=3)*	*Qualified nephrologists for smoking cessation (N=13)*
2020	3079	206 (6.69)	8 (3.88)	0	0	1
2021	3550	344 (9.69)	46 (13.37)	17 (4.94)	2	4
2022*	4285	421 (9.82)	38 (9.02)	20 (4.75)	2	4

* COVID-19 pandemic outbreak in Taiwan in 2022 and lacking enough medication of Champix. Pre-ESRD P4P: pay-for-performance (P4P) program for Pre-end-stage renal disease care education. CKD: chronic kidney disease.

Furthermore, the number of smokers among Pre-ESRD patients also exhibited an upward trend. In 2020, there were 206 smokers (6.7%), which increased to 344 (9.7%) in 2021 and further to 421 (9.8%) in 2022. The number of identified smokers among Pre-ESRD patients as well as the rate of identification increased.

Prior to implementing the integrated OSTC-Pre-ESRD program in 2020, the proportion of smoking cessation among Pre-ESRD patients was only 3.88% (n=3). However, after the implementation of the integrated program, the proportion of smoking cessation significantly increased on a yearly basis, reaching 13.4% (n=46) in 2021 and 9.0% (n=38) in 2022 (Table 3).

In addition to the higher proportion of found smokers and smoking cessation, the number of on-site smoking cessations (OMSC-pre-ESRD program) also showed a substantial increase after the implementation of the integrated system. There were no recorded on-site smoking cessations in 2020, but this number rose to 17 (5.0%) in 2021 and further to 20 (4.8%) in 2022.

In this QI study, we discovered the significance of more motivation among both smokers and medical staff members, including CKD educators and nephrologists. To address this, we formed a core team to develop a stakeholder mapping and an action plan. The outcomes of our efforts included an increase in the number of qualified CKD educators and nephrologists dedicated to promoting smoking cessation for patients. We successfully integrated our electronic medical record systems with both the smoking cessation and Pre-ESRD care program, allowing for routine screening of patients under Pre-ESRD care to determine their smoking status. The implementation of an on-site smoking cessation program greatly enhanced the access and availability of smoking cessation services. Despite challenges such as the COVID-19 pandemic and a shortage of Champix medication in 2022, the number of CKD patients significantly increased compared to the pre-QI period of 2020. This study represents the first detailed examination of the QI process for smoking cessation in patients with Pre-ESRD.

The strength of this QI is the improved access and availability of smokers with CKD. Known factors associated with the success of smoking cessation are being young, female^25^, and low education or low socioeconomic status^26,27^. As for smoker-related factors, smoking service characteristics have an independent effect on the success of smoking cessation^28^. According to the ‘transtheoretical model’^29,30^, since smokers’ motivation to quit changes quickly, the waiting time needs to be kept as short as possible. A recent study^31^, reported that immediate smoking cessation support for smokers increases quit rates measured at 3 months later. Some studies are trying to find the best referral way to shorten waiting time and promote successful smoking cessation^32,33^. In our on-site program, there was no need to refer smokers to another location, as they could immediately begin smoking cessation on-site.

The unique features of our OMSC-Pre-ESRD care program are self-identification of smoking status and the compliance of follow-up. Since cigarette smoking is a risk factor for CKD development^34,35^, our nephrologists and CKD educators were required to screen the smoking status of these patients.

Taiwan’s National Health Insurance is a universal and mandatory insurance system that has been in place since 1995. It is a single-payer system and currently provides coverage for over 99% of the population. In 2006, the insurance system implemented a P4P scheme that included indicators for Pre-ESRD care for patients in CKD stages 3b to 5. Our institute in Taiwan has the highest number of cases receiving Pre-ESRD P4P care, and based on our performance, we became the first institute in Taiwan to pass the national review exercise for kidney disease-specific care. As a result, we were invited to speak at the ISQua’s 37th International Conference in 2021, where we presented our experience with Pre-ESRD P4P care, including quality improvement in standard care, risk management, investigation of adverse events, and ensuring patient safety. With our well-designed Pre-ESRD P4P care program, we seamlessly incorporated the OMSC into routine care. Our on-site, immediate, simple, and team-based smoking cessation model exemplifies the spirit of OMSC. We strongly recommend that institutes with robust chronic disease care programs consider adopting our integrated smoking cessation model.

### Limitations

There are several limitations to this study. Firstly, the proportion of smoker patients was low (<5%). This can be attributed to it being the first year of implementing the QI initiative during the COVID-19 outbreak, along with a shortage of Champix supply in the second year. However, as the COVID-19 situation improved significantly by the end of 2022, we anticipate having more opportunities to promote smoking cessation for CKD patients in the future. Secondly, we did not have satisfactory data on rates. While the feedback from CKD educators and nephrologists was positive, and most patients reported shorter waiting times, we plan to collect and report data on satisfaction rates in the third or fourth year of the QI initiative. Thirdly, we lacked data on the success rate of smoking cessation. Although the increase in case numbers was significant compared to the period without the QI intervention, the overall patient numbers were still relatively low to draw statistically robust conclusions. We intend to calculate the success rate in the third or fourth year of the QI initiative. Lastly, it is important to note that this study is observational in nature, and the intervention was not conducted in a blinded manner.

## CONCLUSIONS

After implementing this QI intervention with our integrated OMSC-Pre-ESRD program, a greater number of our smoker patients successfully quit smoking. This QI program emphasized the significance of improved access and availability for smoking cessation.

## Data Availability

The data supporting this research cannot be made available for privacy or other reasons.
