# Supplementary material for: A quality improvement initiative for patients with chronic kidney disease to promote their smoking cessation
Source: Tob Induc Dis. 2023 Oct 9;21:127. doi: 10.18332/tid/170626 (PMC10561597; doi:10.18332/tid/170626)
Supplement: Supplementary file 1 [file TID-21-127-s1.pdf]

Supplementary file

Figure S1. Dashboard of Ottawa model for smoking cessation in Pre-ESRD care (screenshot in our system in 2023)

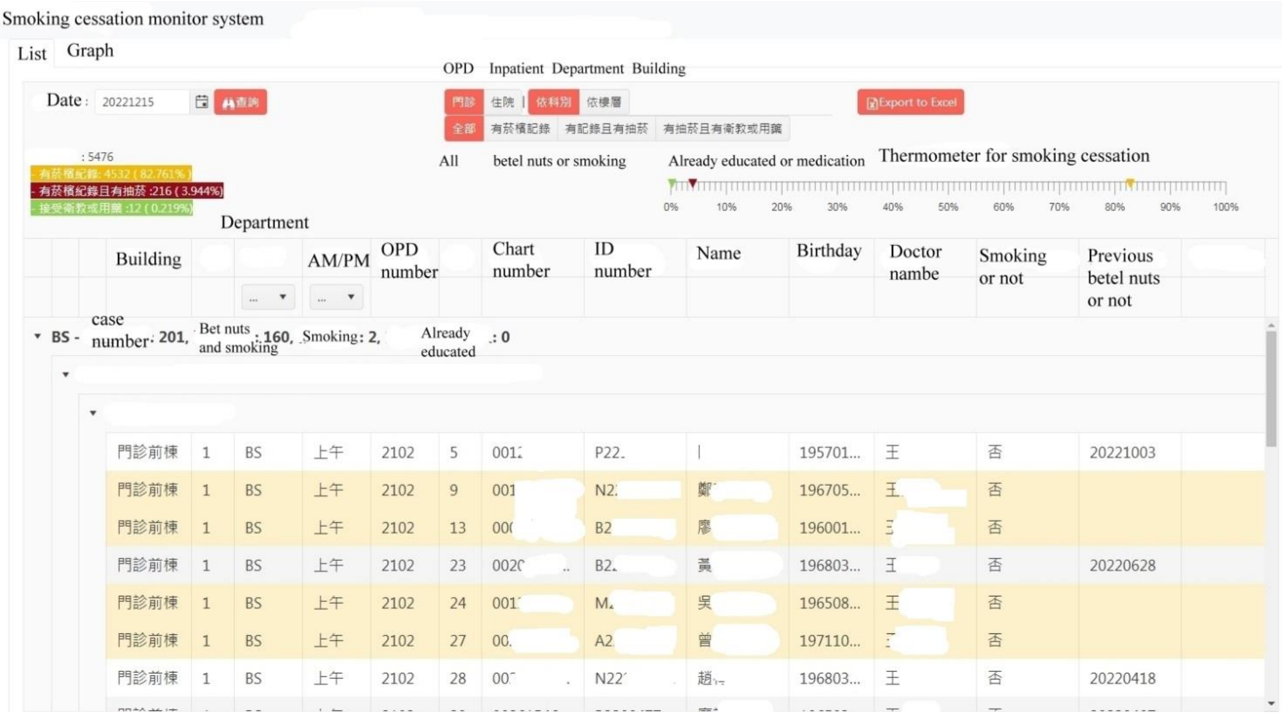

Pre-ESRD: pre-end-stage renal disease.

Figure S2. Integration of OMSC program into electronic medical records (screenshot in our system in 2023)

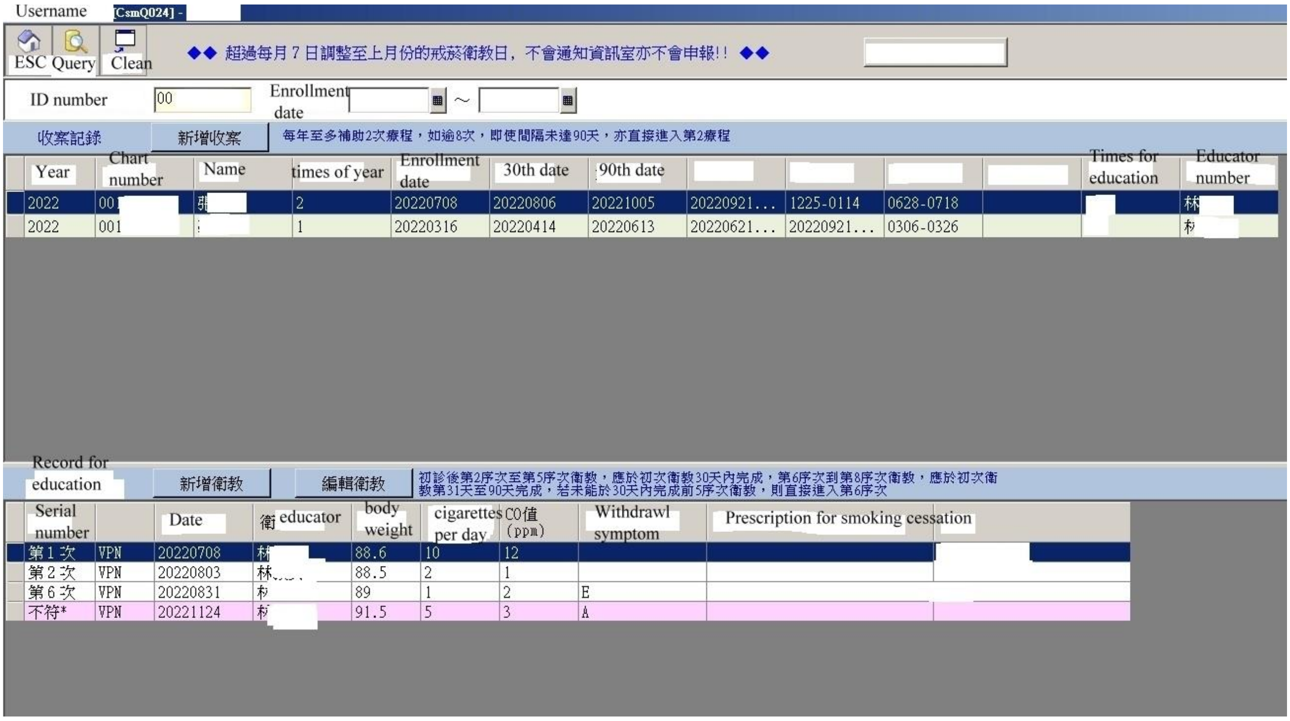

OMSC: Ottawa model for smoking cessation.

© 2023 Chen C.H. et al.
